# Warmer Temperatures Affect the *in situ* Freezing Resistance of the Antarctic Vascular Plants

**DOI:** 10.3389/fpls.2018.01456

**Published:** 2018-10-08

**Authors:** Angela Sierra-Almeida, Lohengrin A. Cavieres, León A. Bravo

**Affiliations:** ^1^Departamento de Botánica, Facultad de Ciencias Naturales y Oceanográficas, Universidad de Concepción, Concepción, Chile; ^2^Instituto de Ecología y Biodiversidad, Santiago, Chile; ^3^Departamento de Ciencias Agronómicas y Recursos Naturales, Facultad de Ciencias Agropecuarias y Forestales and Center of Plant, Soil Interactions and Natural Resources Biotechnology, Scientific and Technological Bioresource Nucleus, Universidad de La Frontera, Temuco, Chile

**Keywords:** Antarctica, climate change, *Colobanthus quitensis*, *Deschampsia antarctica*, freezing events, LT_50_, photoinactivation, warming

## Abstract

Although positive effects on growth and reproduction of Antarctic vascular plants have been reported under warmer temperatures, it could also increase the vulnerability of these plants to freezing. Thus, we assessed *in situ* whether warming decreases the freezing resistance of *Colobanthus quitensis* and *Deschampsia antarctica*, and we compared the level and mechanism of freezing resistance of these species in the field with previous reports conducted in lab conditions. We assessed the freezing resistance of *C. quitensis* and *D. antarctica* by determining their low temperature damage (LT_50_), ice nucleation temperature (NT) and freezing point (FP) in three sites of the King George Island. Plants were exposed during two growing seasons to a passive increase in the air temperature (+W). +W increased by 1K the mean air temperatures, but had smaller effects on freezing temperatures. Leaf temperature of both species was on average 1.7K warmer inside +W. Overall, warming decreased the freezing resistance of Antarctic species. The LT_50_ increased on average 2K for *C. quitensis* and 2.8K for *D. antarctica.* In contrast, NT and FP decreased on average *c.* 1K in leaves of warmed plants of both species. Our results showed an averaged LT_50_ of -15.3°C for *C. quitensis*, and of -22.8°C for *D. antarctica*, with freezing tolerance being the freezing resistance mechanism for both species. These results were partially consistent with previous reports, and likely explanations for such discrepancies were related with methodological differences among studies. Our work is the first study reporting the level and mechanisms of freezing resistance of Antarctic vascular plants measured *in situ*, and we demonstrated that although both plant species exhibited a great ability to cope with freezing temperatures during the growing season, their vulnerability to suffer freezing damage under a warming scenario increase although the magnitude of this response varied across sites and species. Hence, freezing damage should be considered when predicting changes in plant responses of *C. quitensis* and *D. antarctica* under future climate conditions of the Antarctic Peninsula.

## Introduction

Antarctica is the coldest and windiest landmass on Earth (Robinson et al., 2003). Mean air temperatures in the coastal zone of the Antarctic Peninsula and adjacent islands (also called Maritime Antarctica) seldom exceed 0 or +5°C during the summer (Smith, 2003), with daily temperature ranges from -10 to +15°C for the same period (Convey, 2013). Thus, Antarctic plants are constantly dealing with low temperatures, even during the growing season (Convey, 1996; Convey et al., 2014). For this reason, low temperature stress seems to be part of the explanation for its lower plant species diversity compared to the Arctic (Convey, 2006). Only two vascular plants have been able to establish natural populations in the maritime Antarctica: The pearlwort *Colobanthus quitensis* (Kunth) Bartl. (Caryophyllaceae) and the hair grass *Deschampsia antarctica* Desv. (Poaceae) (Smith, 2003).

The constant low temperatures that characterize the growing period in the Maritime Antarctica are likely to be near the minimum thresholds for many physiological processes. This suggests that in the context of climate change, small increments in the temperature experienced by plants in this environment will have a relatively greater biological impact than the same increment experienced in a less extreme environment (Convey, 2001, 2006). During the last part of the past century air temperatures in the Antarctic Peninsula increased at a faster rate than the rest of Antarctica and the globe (Vaughan et al., 2003; Turner et al., 2014). This warmer climate caused longer growing seasons with higher temperatures, ice retreats and higher frequency of rains, which promoted the expansion and increase of population sizes and numbers of *C. quitensis* and *D. antarctica* along the Peninsula (Fowbert and Smith, 1994; Gerighausen et al., 2003; Torres-Mellado et al., 2011; Cannone et al., 2016). Apparently, warmer temperatures favored plant growth and reproduction by providing more favorable thermal conditions for different physiological processes (e.g., photosynthesis), but also by increasing plant nutrient availability via stimulation of soil microbial activity in N-cycling (Wasley et al., 2006; Yergeau et al., 2012). However, these positive effects of warmer temperatures in Antarctica could be negligible because warming can decrease plant freezing survival (Inouye, 2000).

The ability to survive freezing temperatures (i.e., freezing resistance) is highly related to the ambient temperature that plants experience (Beck et al., 2004; Bannister et al., 2005). Thus, warmer daytime temperatures due to climate change may decrease the ability of plant to survive freezing conditions. This is particularly important for high-latitude and -elevation plants to resist freezing temperatures, where it has been shown that warming turned even the most freezing-resistant species more vulnerable to damage by freezing (Loveys et al., 2006; Woldendorp et al., 2008; Rixen et al., 2012). For example, Marchand et al. (2006) reported a reduction of plant performance (i.e., leaf relative chlorophyll content, maximum efficiency of photosystem II, stomatal conductance) of four Arctic plant species after they were exposed to consecutive heat waves. Apparently, warmer conditions reduced cold acclimation of plants, resulting in damage after the exposure to the natural low temperatures of the Arctic. Likewise, Sierra-Almeida and Cavieres (2010) reported that *in situ* warmer temperatures decreased on average 4K the freezing resistance of seven alpine species of central Chilean Andes. Although Turner et al. (2016) reported that warming in the Antarctic Peninsula (AP) has stopped in the last decade; they warned that new warming episodes are likely to occur in the future. In addition, Lee et al. (2017) pointed out that the recently paused warming observed in the AP is a consequence of short-term natural climate variability and that a new warming phase will be observed across the AP. Thus, to assess whether warmer temperatures reduce the ability of Antarctic plants to resist freezing is crucial to predict their vulnerability to future warming events.

Chronically low temperatures of the Antarctica suggest that *C. quitensis* and *D. antarctica* are morphological and physiologically adapted to cope with these stressful conditions. Overall, plants have two physiological mechanisms of freezing resistance: avoidance and tolerance. Freezing avoidance (FA) prevents the ice formation through freezing point depression or by supercooling, meanwhile freezing tolerance (FT) is defined as the ability of plants to survive the extracellular freezing (Larcher, 2003). In the case of Antarctic plants, they have exhibited freezing avoidance and tolerance mechanisms (Bravo et al., 2001; Reyes-Bahamonde, 2013). Molecular and biochemical aspects of the freezing resistance have been studied in these species, especially in *D. antarctica* (e.g., Bravo and Griffith, 2005; Olave-Concha et al., 2005; Piotrowicz-Cieślak et al., 2005; Bravo et al., 2009; Zúñiga-Feest et al., 2009). However, studies dealing with the freezing temperature causing injury to these species are scarce (Bravo et al., 2001; Gianoli et al., 2004; Chew et al., 2012). Studies with laboratory grown plants have reported that the freezing resistance of *C. quitensis* fluctuates between -14 and -4.8°C, whilst for *D. antarctica* it fluctuates between -26.4 and -12°C. Discrepancies among those studies on the level of freezing resistance of Antarctic plants have been attributed to methodological issues. Particularly, the time and temperature that plants were maintained under greenhouse and/or growth chambers before freezing injury assays varied enormously among them. Therefore, *in situ* determinations are required to unveil the real level of freezing resistance of the Antarctic vascular plants and the mechanisms involved. In addition, manipulative field experiments are needed to assess the likely effect of warming in this important trait.

In this study, we conducted a field experiment in the King George Island, where we increased the air temperatures experienced by *C. quitensis* and *D. antarctica* during two growing seasons to assess their *in situ* vulnerability to freezing damage under different thermal conditions. Specifically, our aims were: (1) to assess the *in situ* level of the freezing resistance of *C. quitensis* and *D. antarctica* and whether warming decrease this ability (i.e., leaf NT, FP and/or LT_50_ of warmed plants should occur at higher temperatures than of unwarmed plants); and (2) to compare the level and mechanism of freezing resistance of these Antarctic plants species in the field with previous reports conducted under lab conditions.

## Materials and Methods

### Study Area

**FIGURE 1:**
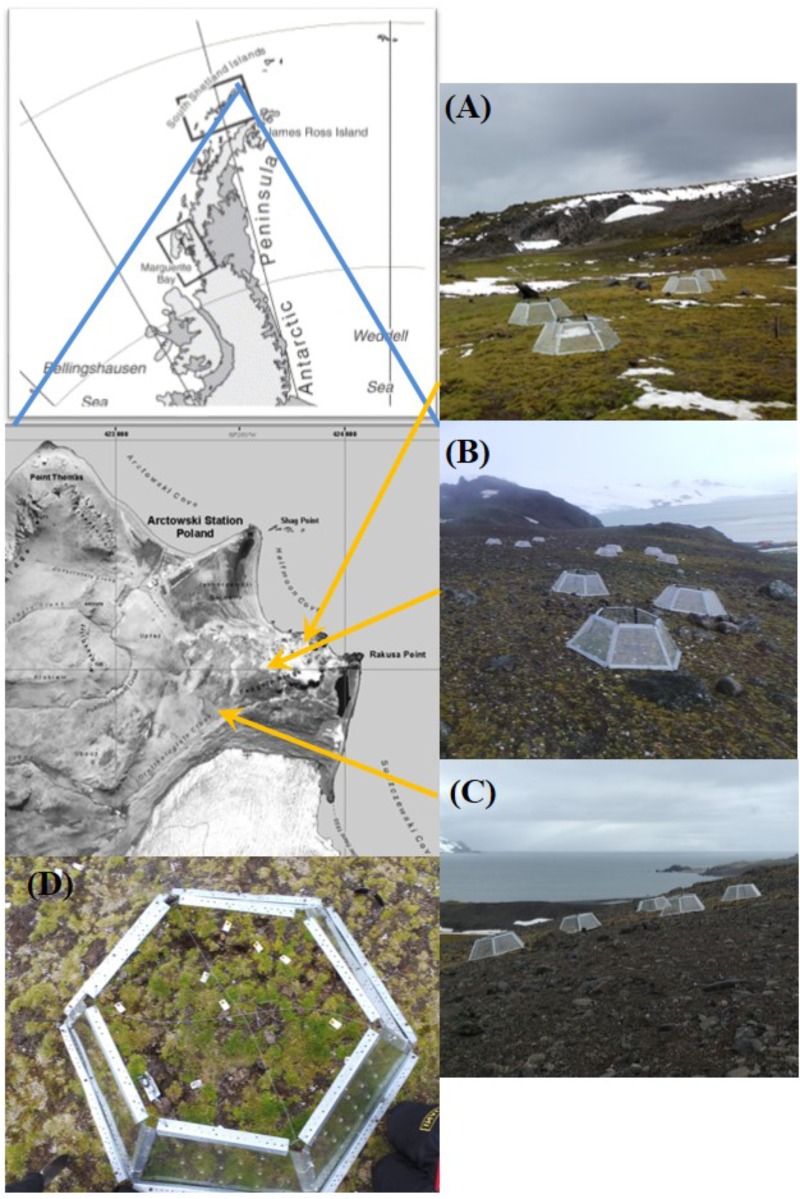
Study area in the King George Island (South Shetland Archipelago) where experimental warming was installed. Photographs correspond to **(A)** Site 1, nearest the beach; **(B)** Site 2, and **(C)** Site 3; **(D)** showed several individuals of *C. quitensis* inside an OTC (see Materials and Methods sections for site descriptions and experimental design).

This study was carried out in the King George Island, South Shetland Archipelago, nearby the Henryk Arctowski Polish Station (62°09′ S, 58°28′ W). Plants were obtained from three sites, which differed in soil nutrients, plant cover and relative abundance of the Antarctic vascular species (**Supplementary Table S1** and **Figure 1**). Site 1 (62° 9′43.33″S; 58°27′58.80″W) was located near the beach, about 90 m from the coast line thus receiving sea spray, plant cover is >90% and vegetation is dominated by *D. antarctica.* This site receives great inputs of guano and feces because of the activity of sea birds and mammals (see **Supplementary Table S1** for soil nutrient contents). Site 2 (62° 9′49.15″S; 58°28′9.60″W) was located 200 m distant of the site 1, plant cover is 100% and it is dominated by a compact and continuous moss carpet where C. *quitensis* and *D. antarctica* are growing interspaced (Cavieres et al., 2018). This site seems to be favorable for plant growth because of well drained soils and nutrient availability (**Figure 1** and **Supplementary Table S1**). Site 3 (62° 9′52.90″S; 58°28′21.31″W) is a typical fellfield located 550 m from the beach and at 30 m a.s.l. Plant cover is <10%, and the scarce vegetation is dominated by lichens and only isolated individuals of *C. quitensis* and *D. antarctica* species are present across a stony and rocky soil matrix (**Figure 1**).

The growing season in the study area usually starts with the snowmelt in December and finishes in March with the first snowfall. During two growing periods we collected microclimatic data (83 and 55 days for the first and second growing season, respectively), period ever which the daily mean air temperature was 1.8°C, with mean maximum and minimum temperatures of 4.7 and -0.4°C, respectively, with night freezing temperatures occurring frequently during the entire growing seasons (**Table 1**). Precipitation occurs as rain in summer, with estimations that range from 350 to 750 mm (Green et al., 2007).

**Table 1:** Thermal conditions of Antarctic plants exposed to experimental warming during two growing seasons in the King George Island, Maritime Antarctica (Antarctic Peninsula).

		Site 1		Site 2		Site 3	
	Variable	-W	+W	-W	+W	-W	+W
**2014**	Length of the growing season (days)	77		89		83	
	Mean air temperature (T_mean_, °C)	1.3 ± 0.1	2.7 ± 0.1^∗^	0.9 ± 0.1	2 ± 0.1^∗^	-	-
	Maximum air temperature (T_max_, °C)	4.5 ± 0.4	9.1 ± 0.6^∗^	3.9 ± 0.3	6.6 ± 0.4^∗^	-	-
	Minimum air temperature (T_min_, °C)	-1 ± 0.2	-0.8 ± 0.2	-1.3 ± 0.2	-0.8 ± 0.2	-	-
	GDD_0_ (°C day^-1^)	116.6	288.5^∗^	115.1	254.5^∗^	-	-
	Frequency of freezing events (F_freq_, %)	77	73	80.9	75.3	-	-
	Intensity of freezing events (F_int_, °C)	-1.7 ± 0.2	-1.6 ± 0.2	-1.9 ± 0.2	-1.5 ± 0.1	-	-
	Absolute min temperature (°C)	-6.5	-4.9	-6.8	-5.8		
	Duration of freezing events (F_dur_, h)	11.9 ± 0.8	9.9 ± 0.8	17.6 ± 0.8	13.1 ± 0.6^∗^	-	-
	Minimum soil temperature (°C)	3.4 ± 0.2	4.8 ± 0.3^∗^	1.7 ± 0.1	2.4 ± 0.1^∗^	3.1 ± 0.1	4.3 ± 0.1^∗^
	Leaf temperature of *C. quitensis* (°C)	4 ± 0.1	4.9 ± 0.1^∗^	2.3 ± 0.1	3.8 ± 0.1^∗^	-	-
	Leaf temperature of *D. antarctica* (°C)	3.7 ± 0.1	5.3 ± 0.1^∗^	2.4 ± 0.1	4.9 ± 0.2^∗^	-	-
**2015**	Length of the growing season (days)	53		55		55	
	Mean air temperature (T_mean_, °C)	2.7 ± 0.1	3.7 ± 0.1	2.1 ± 0.1	2.9 ± 0.1	2.1 ± 0.1	3.1 ± 0.1
	Maximum air temperature (T_max_, °C)	5.3 ± 0.4	9.2 ± 0.6^∗^	4.9 ± 0.4	7.8 ± 0.5^∗^	5.1 ± 0.4	8.4 ± 0.5^∗^
	Minimum air temperature (T_min_, °C)	0.6 ± 0.3	0.3 ± 0.3	0 ± 0.3	-0.1 ± 0.3	-0.1 ± 0.3	-0.02 ± 0.3
	GDD_0_ (°C day^-1^)	152.2	242.4^∗^	128.2	206.5^∗^	138.3	220.5^∗^
	Frequency of freezing events (F_freq_, %)	27.5	37.3	47.3	49.1	60	41.8
	Intensity of freezing events (F_int_, °C)	-2.2 ± 0.4	-1.9 ± 0.3	-1.8 ± 0.3	-1.8 ± 0.3	-1.4 ± 0.4	-1.8 ± 0.3^∗^
	Absolute min temperature (°C)	-5	-5.6	-6.2	-6.2	-6.4	-5.8
	Duration of freezing events (F_dur_, h)	10.6 ± 2.5	10.1 ± 2	11.7 ± 2.4	10.7 ± 2.2^∗^	11.2 ± 2.3	11.4 ± 1.8
	Minimum soil temperature (°C)	3.5 ± 0.3	4.4 ± 0.3^∗^	1.6 ± 0.2	2.1 ± 0.2	2.5 ± 0.2	2.4 ± 0.2
	Leaf temperature of *C. quitensis* (°C)	-	-	3.4 ± 0.1	3.8 ± 0.1	3.2 ± 0.1	5.1 ± 0.1^∗^
	Leaf temperature of *D. antarctica* (°C)	-	-	3 ± 0.1	4.7 ± 0.2^∗^	3.8 ± 0.1	5.7 ± 0.1^∗^

*Values correspond to mean ± SE (n = 1), and they are shown for natural temperature conditions (-W) and warming (+W) at three sites in the study area. Asterisks indicate significant differences between natural and warm conditions (χ^2^, P < 0.05).*

### Plant Species

Studied species were the pearlwort *C. quitensis* (Kunth) Bartl. (Caryophyllaceae) and the hair grass *D. antarctica* Desv. (Poaceae). *C. quitensis* is a long-lived perennial herb. It forms low, compact, discrete cushions with densely packed shoots and a log taproot (Greene and Holtom, 1971). Its geographical distribution comprises from Mexico and from the Andes mountains of Ecuador down to *c.* 68°S in the Maritime Antarctica (Moore, 1970). Despite its wide latitudinal distribution, this species inhabits sites with similar conditions, characterized by sparsely vegetated, sheltered, moist, and well-drained mineral soils (Smith, 2003). *D. antarctica* is a long-lived perennial herb that forms low, caespitose shallow-rooted tufts (Greene, 1964; Moore, 1979). *D. antarctica* distributes from central Chile and Argentina (33°S) to the Terra Firma Islands southwestern Antarctic Peninsula (68°S; Smith and Poncet, 1987). In Antarctica this species colonize habitats ranging from mineral to organic soils, from well drained to waterlogged areas, and from nutrient-deficient to highly nutrient-enriched habitats (Smith, 2003).

### Experimental Design

In December 2013, on each site we selected seven plant individuals of *C. quitensis* and *D. antarctica*. On each individual, we placed a hexagonal Open Top Chamber (thereafter OTC), similar to those used in the International Tundra Experiment (ITEX). Each OTC was made with transparent Plexiglass^®^walls of 40 cm height, 115 cm in basal diameter, and reinforced with aluminum profiles. OTCs walls were punched with 25 holes of 1.5 cm diameter each to allow some wind to pass through and hence avoid an excessive increase in air temperature. OTCs were secured to the ground with ropes to avoid being moved and/or destroyed by the strong winds. Another seven individuals per species were randomly selected at 2 m distant from the nearest OTC. These individuals were growing under natural temperatures conditions. Hence, we obtained two experimental conditions with 7 replicates each: warmed (+W) and unwarmed control plants (-W) repeated in three sites. The spatial arrangement of both OTC and control plots was random, taking care that distance between OTC is enough to avoid any possible effects of OTCs on the neighboring control plots by affecting wind or snow deposition. Although the use of passive warming systems such as OTC has been controversial (e.g., Kennedy, 1995; De Boeck et al., 2012), some authors arguably consider that OTCs are a reasonable analog of regional warming for remote areas such as polar habitats (Hollister and Webber, 2000; Bokhorst et al., 2013).

Microclimatic conditions were monitored in warming and control plots during two growing seasons (**Table 1**). For this, a weather station was installed on each site (2 units HOBO^®^U-30 Station, Onset Computer Co., Bourne, MA, United States; 1 Em50 Data Logger, Decagon Devices Inc., Pullman, WA, United States). Air and soil temperature sensors were placed at 5 cm (*n* = 1) above and 5 cm below (*n* = 1) ground surface, and temperature conditions were recorded every hour. In addition, leaf temperature was measured for warmed and unwarmed plants of *C. quitensis* and *D. antarctica*. For this, each leaf temperature sensor was placed beneath a leaf (*n* = 1) and were connected to the weather station programmed to record temperature every hour. Missing leaf temperatures data in site 1 during 2015 were absent because sensors were destroyed. A similar situation occurred with air and leaf temperature sensors in site 3 during 2014. Due to logistic limitations to access the study area, weather stations were installed and uninstalled in the field for each growing season. Thus, records of microclimatic conditions in the **Table 1** started in December 10th 2013 and finished in March 8th 2014 for the first growing season. For the second growing season records started in January 10th (sites 2 and 3) and 13th (site 1) and finished in March 4th 2015.

Air temperature data were used to estimate growing degree days (GDDs; McMaster and Wilhelm, 1997). GDDs were used as a measure of the accumulated amount of heat (in °C) above a base temperature to represent a cumulative index of the energy available to growing plants, according to the formula:

GDD=[[(maximumdailytemperature+minimumdailytemperature)/2]]−basetemperature

The daily GDDs were summed per each entire growing season. We used 0°C as a conservative base growing temperature (the temperature above which plants can perform metabolic functions, e.g., photosynthesis, cell elongation), because plants from cold climate generally vary in their absolute base growing temperature, and this value encompasses this variability (Körner, 2011).

### Freezing Resistance Determinations

#### Plant Material Collection

We collected seven plant samples replicates for each species, experimental condition and site, excepting by the site 3 where we collected six replicates. Plant samples corresponded to complete individuals with at least seven modules (small rosettes or tillers). We collected all plant material between 11:00 AM and 12:00 PM. Plant samples were placed in plastic boxes with belowground organs wrapped in wet paper to prevent changes in tissue water content and mechanical damage. Samples were then transported to a field laboratory at the Polish Scientific Station, less than 10 min away from the study sites. We kept plant samples outdoor but protected from wind until freezing resistance determinations were performed within 24 h of collection, which were carried out between February 25th and March 5th 2015.

#### Low Temperature Damage

For each species, experimental condition and site, we estimated the freezing temperature producing 50% damage (LT_50_). For this, we selected and detached six rosettes/tillers from different plant samples, and they were separated into six subsamples. One subsample was used as control and stored at 2°C and darkness during 24 h. Remaining five subsamples were separately placed in a small plastic bag, which was then placed in a larger plastic bag with a weight to ensure that each subsample was submerged in a cryostat (F25-ME, Julabo Labortechnik GmbH, Germany) with antifreeze solution (Polycool Mix 25, PolyScience, IL, United States). Cryostat was cooled previously at five different target temperatures: -8, -12, -16, -20 and -25°C. All subsamples were transferred from outdoor to the cryostat and incubated during 2 h to reach homogeneous leaf temperatures. After that freezing treatment, subsamples were removed from the cryostat and placed back into cold room, under darkness and at 2°C during 24 h for thawing. In most studies dealing with plant freezing resistance, samples are cooled gradually to determine LT_50_ (2–5 K h^-1^; e.g., Hekneby et al., 2006; Ladinig et al., 2013; Briceño et al., 2014). However, given that cooling rates used by previous studies dealing with freezing resistance of Antarctic vascular plants varied from 1 to 17 K h^-1^ (Bravo et al., 2001; Gianoli et al., 2004; Chew et al., 2012), it makes impossible to find a consensus cooling rate for comparative purposes. Although sudden cooling can lead to increased tissue damage owing to anomalous water diffusion and ice crystal formation (Guy, 2003; Wisniewski et al., 2014), our procedure induced similar plant damage as a cooling rate of 16 K h^-1^ (see Pescador et al., 2018 for details of cooling rates assay), enabling to assess cooling directly as proxy for natural and immediate freezing exposure (Larcher et al., 2010).

Leaf damage was assessed as percentage of photoinactivation (*PhI*) of the photosystem II as described by Larcher (2000). For this, we measured the ratio of variable to maximum fluorescence (*Fv/Fm*) of dark-adapted leaf by using a chlorophyll fluorometer (MINI-PAM, Walz, Germany). LT_50_ corresponds to the temperature at which *PhI* reaches a 50% value in subsamples, and was determined by linear interpolation using the temperature of the highest *PhI* of <50% and the temperature of the lowest *PhI* of >50% (Bannister et al., 2005). *PhI* was chosen because it measures changes in photosynthetic performance that correlates very well with direct measurements of tissue damage (i.e., visual freezing injuries and vital stain; Boorse et al., 1998; Neuner and Buchner, 1999) and because is an easy, rapid and cheap method to work in areas with difficult logistic as it is the Antarctica.

#### Thermal Analyses

A small module (rosette or tiller) was removed from each of six-seven plant samples taken from each species, experimental condition and sites. Each module was attached to a thermocouple (Gauge 30 copper-constantan thermocouples; Cole Palmer Instruments, Vernon Hills, IL, United States), and immediately enclosed in a small, tightly closed cryotube. The cryotubes were placed in a cryostat (F25-ME, Julabo Labortechnik GmbH, Germany), and the temperature was decreased from 0 to -20°C, at a cooling rate of 2 K h^-1^. The temperature of individual module was monitored every second with a Personal Daq/56 multi-channel thermocouple USB data acquisition module (IOtech, Cleveland, OH, United States). The sudden rise in leaf temperature (exotherm) produced by the heat released during the extracellular freezing process was used to determine two variables: the ice nucleation temperature (NT), which corresponds to the lowest temperature before the exotherm, indicating the onset of ice crystal formation, and the freezing point (FP), the highest point of the exotherm, indicating the freezing of water in the apoplast, including symplastic water driven outward by the water potential difference caused by the apoplastic ice formation (Larcher, 2003). We chose this cooling rate because it is the same used by previous studies where thermal analyses were carried out (Bravo et al., 2001; Reyes-Bahamonde, 2013).

### Statistical Analyses

Differences in air (i.e., mean, maximum, minimum, intensity, and duration of freezing events) and leaf temperatures between warming and control conditions were assessed by Chi square (*χ*^2^) tests. Differences in the effect of warming and site on LT_50_, NT and FP were assessed by using Factorial ANOVAs as well (See details in **Supplementary Table S2**). Differences between NT and LT_50_ in determining freezing resistance mechanisms for each species and experimental conditions were assessed with *t*-tests. Data were checked for normality before analyses.

## Results

### Microclimatic Conditions

Air, soil and leaf temperatures during both growing seasons were affected by warming (**Table 1**). Although minimum air temperatures (T_min_) and the intensity of freezing events (F_int_) were similar between +W and -W conditions (average T_min_ ranged from -13 to 0.6°C and average F_int_ was -1.8°C in both growing periods), the frequency (F_freq_) and duration of freezing events (F_dur_) tended to be lower and of shorter duration inside +W plots (**Table 1**). The maximum air temperature (T_max_) was consistently higher inside +W plots. For example, in 2014 +W increased 4.6 and 2.7°C the air T_max_ in the sites 1 and 2, respectively. This T_max_ increase was of 3.9, 2.9, and 3.3°C in the sites 1, 2, and 3 in 2015. Regarding the growing degree days above 0°C (GDD_0_), in 2014 GDD_0_ were 59.6 and 54.8% greater in +W than in –W conditions in the sites 1 and 2, respectively (**Table 1**). In 2015, +W increased GDD_0_ on average 37% in the three sites (**Table 1**).

### Freezing Resistance in the Field Under Warming Scenario

**FIGURE 2:**
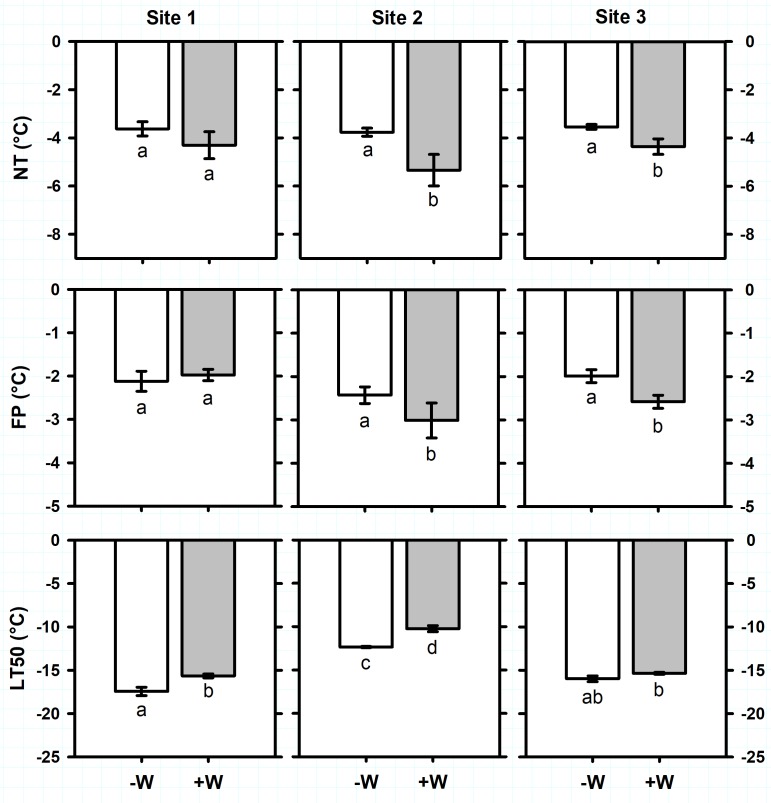
Freezing resistance of *Colobanthus quitensis* measured in plants growing in three sites in the King George Island. Freezing parameters measured were: NT, ice nucleation temperature (°C); FP, freezing point (°C); and LT_50_, freezing temperature producing 50% photoinactivation (°C). Values correspond to mean ± SE (*n* = 6–7). Treatments were: –W, plants under natural temperature conditions; +W, plants under warm temperature conditions. Less negative values indicate higher freezing resistance. Significant differences between natural and warm conditions are shown as different lowercases (*P* < 0.05).

**FIGURE 3:**
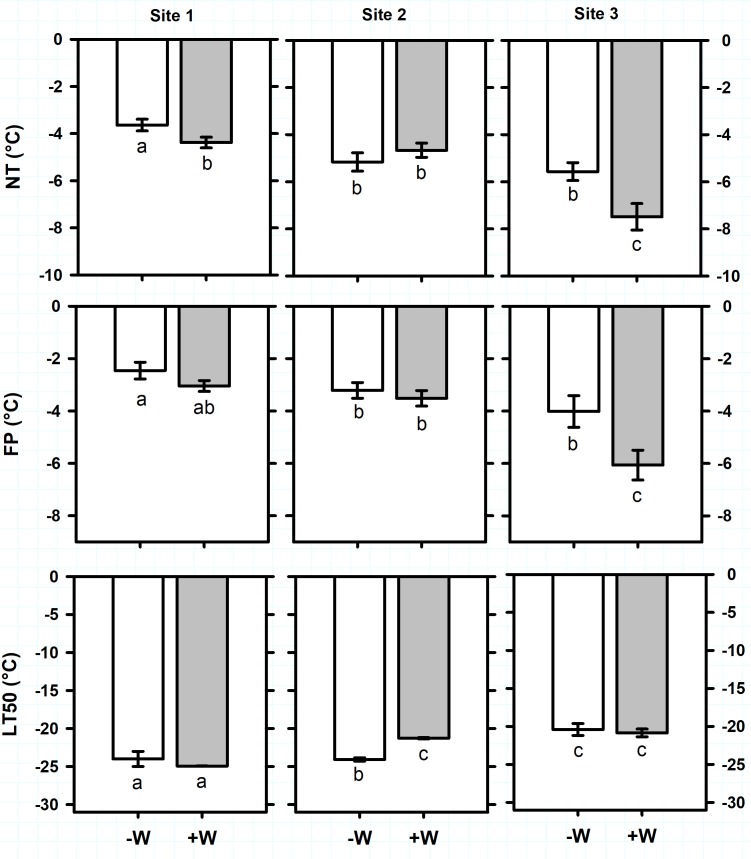
Freezing resistance of *Deschampsia antarctica* measured in plants growing in three sites in the King George Island. Freezing parameters measured were: NT, ice nucleation temperature (°C); FP, freezing point (°C); and LT_50_, freezing temperature producing 50% photoinactivation (°C). Values correspond to mean ± SE (*n* = 6–7). Treatments were: –W, plants under natural temperature conditions; +W, plants under warm temperature conditions. Less negative values indicate higher freezing resistance. Significant differences between natural and warm conditions are shown as different lowercases (*P* < 0.05).

Antarctic plant species exhibited different ranges of freezing resistance in the field (**Supplementary Table S3**). Considering all sites together, average of ice Nucleation Temperature (NT) and Freezing Point (FP) of *C. quitensis* were -3.7 ± 0.1°C and -2.2 ± 0.1°C, respectively (**Figure 2**). Freezing temperature producing 50% photoinactivation (LT_50_) ranged from -12.4 ± 0.1°C in site 2 to -17.4 ± 0.5°C in site 1 (**Figure 2**; F_2,32_ = 172.5, *P* < 0.0001). For *D. antarctica*, NT ranged from -3.6 ± 0.3°C in site 1 to -5.4 ± 0.3°C in sites 2 and 3 (**Figure 3**; F_2,34_ = 24.1, *P* < 0.0001). Similarly, FP ranged from -2.5 ± 0.3°C in site 1 to -4 ± 0.6°C in site 3 (**Figure 3**; F_2,32_ = 12.2, *P* < 0.001). In contrast, LT_50_ decreased (more negative) from -20.4 ± 0.8°C in site 3 to -24 ± 0.5°C in sites 1 and 2 (**Figure 3**; F_2,34_ = 22.7, *P* < 0.0001). It seems noteworthy that NT were sharply higher than LT_50_ on *C. quitensis* and *D. antarctica* at any site, suggesting that both species are able to tolerate ice formation within their leaf tissues. Thus, the mechanism of freezing resistance did not vary with site and was freezing tolerance for both *C. quitensis* and *D. antarctica* (**Supplementary Table S3**).

Antarctic plants varied their vulnerability to suffer freezing damage with warming (**Supplementary Table S3**). For *C. quitensis*, warming (+W) increased (less negative temperature) on average 2K the LT_50_ of plants growing in sites 1 and 2 (**Figure 2**; *F*_1,32_ = 34, *P* < 0.0001). In contrast, +W decreased on average 1.2K the NT (*F*_1,32_ = 6.8, *P* = 0.014) and 0.6K the FP (*F*_1,32_ = 2.6, *P* = 0.012) of plants growing at sites 2 and 3 (**Figure 2**). For *D. antarctica*, +W effects on freezing resistance varied with site (**Figure 3**). For example, LT_50_ increased 2.8K with +W but only on plants from site 2 (*F*_2,34_ = 6.7, *P* = 0.003). In contrast, +W decreased 0.8 and 1.9 K the NT of plants growing in sites 1 and 3 (*F*_1,34_ = 5.4, *P* = 0.026), respectively, but no effects on NT were observed in plants at site 2 (**Figure 3**). Similarly, FP of *D. antarctica* inside +W occurred at temperatures 2K more negative than control plants but only at site 3 (*F*_1,34_ = 8.4, *P* = 0.007). Despite the opposite effects of warming on NT and LT_50_ of Antarctic plants, both *C. quitensis* and *D. antarctica* were always classified as freezing tolerant species (**Supplementary Table S3**).

## Discussion

Sufficient levels of resistance to freezing temperatures during the summer is key for the survival, growth and reproduction of *C. quitensis* and *D. antarctica* in the Maritime Antarctica (Cavieres et al., 2016). Paradoxically, the regional warming that promote the growth and reproduction of these species (Cannone et al., 2016) could reduce their survival ability making even the best cold adapted plants more susceptible to damage by freezing temperatures (Woldendorp et al., 2008; Ball et al., 2011). As far as we are aware, our work is the first study reporting the level and mechanisms of freezing resistance of Antarctic vascular plants measured *in situ*, and we demonstrated that both plant species exhibited a great ability to cope with freezing temperatures during the growing season. Nonetheless, increases in the temperatures experienced by plants during the growing season changed this functional trait suggesting increases in their vulnerability to suffer freezing damage under warmer temperature scenarios.

Overall, warmer conditions decreased the freezing resistance of both Antarctic species. That is, LT_50_ occurred at higher (less negative) temperatures in warmed plants of *C. quitensis* and *D. antarctica* (**Supplementary Table S3** and **Figures 2**, **3**). The LT_50_ increased on average 2K for *C. quitensis* and 2.8K for *D. antarctica*, despite mean air temperatures increased only by 1K with OTCs. This suggests that further increases in ambient temperature, as those projected in future climate scenarios could lead to greater changes in this plant functional trait. Whilst LT_50_ increases in warmer conditions were observed at all sites for *C. quitensis*, for *D. antarctica* it showed site specific responses (**Supplementary Tables S2, S3**). These results highlight two aspects that have to be considered. First, *C. quitensis* would be more vulnerable to freezing damage than *D. antarctica* with warmer conditions. Several studies have reported that the Antarctic vascular plants respond differently to warmer conditions (Xiong et al., 2000; Cannone et al., 2016). For example, whereas in *D. antarctica* no effects of warming on leaf carbon gain and plant growth has been observed, in *C. quitensis* warmer temperatures promoted both plant traits (Sáez et al., 2018). Our microclimatic data showed that although freezing temperatures events were frequent and of long duration during the Antarctic summer, the intensity of those events were relatively mild (average of -2°C, with absolute records *c.* -7°C). Previous studies on the climate of the study area have reported summer freezing events of -5 and -7.8°C (Cygan, 1981; Araźny et al., 2013). However, air temperatures suddenly decrease in autumn, with some records of -13°C in April (Araźny et al., 2013). If we considered that snow duration and cover are highly unpredictable in the area because of topography and especially of wind speed (Angiel et al., 2010), Antarctic plants could be frequently exposed to such freezing temperatures during the summer- autumn transition. In that scenario, *D. antarctica* has a temperature safety margin of seldom 10K because warmed plants exhibited an averaged LT_50_ of -22.4°C. However, this safety margin doesn’t exist for *C. quitensis*, which exhibited an averaged LT_50_ of -13.8°C. Although, both species have the ability to cope with summer freezing events in the area, if ambient temperatures continue to rise, as some authors propose (Lee et al., 2017), they might have negative consequences for plant survival of *C. quitensis* but not for *D. antarctica.*

Secondly, a site-dependent response of *D. antarctica* to warming was observed, where warming increased LT_50_ only in plants from site 2. This site is dominated by moss carpets and presents a permanent water-saturated but well drained substrate, abundant in organic matter and N content of 18–40 ppm (Kozeretska et al., 2010; **Supplementary Table S1**). According to substrate preferences of this species, moss carpets is where this species is more abundant and frequent (Casanova-Katny and Cavieres, 2012; Park et al., 2013), and where individual plants grow bigger (Casanova-Katny and Cavieres, 2012; Cavieres et al., 2018). Given that this site presents a greater availability of resources (i.e., water and nutrients) compared to the other sites, and where the presence of neighbors (moss carpets) can ameliorates the harsh climatic conditions (see Casanova-Katny and Cavieres, 2012; Cavieres et al., 2018), the site-dependent LT_50_ response to warming of *D. antarctica* could be attributed to the tradeoff between plant growth and stress resistance, where warmer temperatures are favoring plant allocation to growth. The absence of better soils (site 3), the presence of sea spray and animal disturbance (site 1) and the absence of moss carpets (sites 1 and 3) generate that plants on these sites are constantly dealing with stress, even under warmer conditions.

Contrary to our expectations, NT and FP occurred at lower (more negative) temperatures in leaves of warmed plants of both species. FP and NT depend on specific properties of the plant tissues and may vary according to the cell sap concentration and/or the accumulation of water-binding substances inside the cell (Sakai and Larcher, 1987). NT decreases in plant tissues with small cell sizes, relative low water content, and/or little or no intercellular space for nucleation (Sakai and Larcher, 1987). Sáez et al. (2018) reported that *in situ* warmer temperatures induced changes in morpho-anatomical leaf traits of *C. quitensis* and *D. antarctica* that might relate with the changes in freezing resistance reported here, but further studies are needed to unveil their relation and consequences for the plant freezing resistance. In addition, several studies have reported that water-soluble carbohydrates depress FP, and their accumulation is positively related to abiotic stress survival, which is also the case of these two Antarctic plant species (Bravo et al., 2001; Pastorczyk et al., 2014). We expected that lower FP values contributed to increase the freezing resistance of Antarctic plants, by decreasing their LT_50_. However, this was not the case. This could be related with the fact that +W plants were exposed to warmed temperatures during days but similar cold temperatures as –W plants during the nights. This has two implications. First, plants under +W may be exposed to more frequent freeze/thaw events than plants under -W. This may cause recurrent xylem embolism and cell dehydration (Pearce, 2001), which may induce the warmed plants to keep some freezing avoidance capability such as lower NT and FP than unwarmed plants. It has been observed that the degree of frost hardening may be a function of the number of freezing events (Beck et al., 2004). Second, warmer days imply better conditions for CO_2_ assimilation in the +W treatment (e.g., Sáez et al., 2018) but similar respiration rates during the night on both +W or -W. Then, there is a higher probability that carbohydrates synthesized exceeded carbohydrates respired in +W than in -W treatment with the consequent higher accumulation of non-structural carbohydrate in +W, which can act as compatible solutes reducing the FP, but these putative explanations remain to be elucidated.

Previous studies have measured the freezing resistance of *C. quitensis* and/or *D. antarctica* of plants grown under controlled conditions in the lab. However, there were discrepancies in the level (i.e., LT_50_ values) and mechanism of freezing resistance (i.e., freezing avoidance or tolerance), as well as in their capacity for cold acclimation. For instance, Bravo et al. (2001) reported that *C. quitensis* avoided freezing by supercooling, that non-acclimated plants of *C. quitensis* experienced freezing injury at -4.8°C, when ice nucleation was induced by silver iodine, and its LT_50_ decreased only 1K after cold acclimation at 4°C for 21 days under the same measurement condition. In contrast, LT_50_ of *C. quitensis* decreased from -7 to -15°C with a similar cold-acclimation period without using ice nucleator according to Gianoli et al. (2004) and Reyes-Bahamonde (2013), and they classified *C. quitensis* as a freezing tolerant species (**Table 2**). In the case of *D. antarctica*, all previous studies classified it as a meanly freezing tolerant plant (**Table 3**). According to Bravo et al. (2001), cold-acclimation decreased LT_50_ from -12 to -26.6°C. However, Chew et al. (2012) found that LT_50_ of *D. antarctica* decreased from -12°C in non-acclimated to -17°C in cold-acclimated plants, whilst Reyes-Bahamonde (2013) found that this species exhibited a LT_50_ of -16.5 and -18.4°C in non- and cold-acclimated plants, respectively (**Table 3**).

**Table 2:** Previous studies where freezing resistance of Antarctic plants has been reported.

	*Colobanthus quitensis*		*Deschampsia antarctica*		Freezing injury method
Reference	A	NA	A	NA	
Casanova-Katny, 1997 lab	-	-	-26.4	-14.8	Photoinactivation
Field	-	-	-27	-	Photoinactivation
Bravo et al., 2001	-5.8 (FA)	-4.8 (FA)	-26.6 (FA/FT)	-12 (FA/FT)	Ion leakage
Gianoli et al., 2004	-15	-7	-	-	Plant survival
Chew et al., 2012	-	-	-17	-12	Survival and regrowth
Reyes-Bahamonde, 2013	-14.9 (FT)	-7 (FT)	-18.4 (FT)	-16.5 (FT)	Photoinactivation
This study	-15.3	-	-22.8	-	Photoinactivation

*Values correspond to mean LT_50_ ± SE, obtained for non-acclimated (NA) and cold acclimated plants (A) after 21 days to 2–5°C, excepting for Chew et al. (2012), which corresponded to cold acclimation period of 14 days. Freezing mechanisms are shown between brackets: Freezing Avoidance (FA) and Tolerance (FT).*

**Table 3:** A comparison of two criteria for LT_50_ determinations in *Colobanthus quitensis* and *Deschampsia antarctica*.

Species	Origin	LT_50_ PhI	LT_50_ IonL	Z	*p*
*Colobanthus quitensis*	Arctowski	-7.1 ± 0.5	-6.6 ± 0.4	0.94	0.345
	Punta Arenas	-6.3 ± 0.4	-6.9 ± 0.5	1.48	0.138
	La Parva	-6.7 ± 0.3	-7.3 ± 0.6	2.02	0.053
*Deschampsia antarctica*	Actowski	-19.9 ± 1.5	-20.1 ± 1.9	0.41	0.686

*Plants were collected from different localities, cultivated in growth chambers, and measured in laboratory conditions at the University of Concepción. LT_50_ values (mean ± SE, *n* = 5) were obtained by using two methods: PhotoInactivation (PhI) and Ion Leakage (IonL). Statistical differences were assessed by using Wilcoxon matched paired tests. Followed criteria are described by Flint et al. (1967) and Bannister (2005) respectively.*

Considering all sites together our results showed an average LT_50_ of -15.3 and -22.8°C for *C. quitensis* and *D. antarctica*, respectively, and that both species exhibited freezing tolerance as the mechanism of freezing resistance. In the case of *C. quitensis* our LT_50_ are similar to those previously reported for cold-acclimated plants, which is reasonable considering temperature conditions recorded in the field when the determinations were carried out. For *D. antarctica*, however, LT_50_ were relatively different from previous reports. Likely explanations for the discrepancies in the level and mechanism of freezing resistance of Antarctic vascular plants found here with those previously reported arise from methodological differences among studies. For example the method used to assess freezing injury can lead to important differences. Bravo et al. (2001) estimated LT_50_ of *C. quitensis* and *D. antarctica* from electrolyte leakage by freezing-induced cell lysis, whereas Reyes-Bahamonde (2013) LT_50_ estimations were based on photoinactivation. On the contrary, Gianoli et al. (2004) calculated LT_50_ of *C. quitensis* with plant survival percentage, and Chew et al. (2012) LT_50_ estimations of *D. antarctica* were based on tiller survival from re-growth. It has been reported that photoinactivation method agrees very well with the results obtained by methods that directly measure plant tissue damage (i.e., survival, visual assessment of freezing injuries and vital staining; Boorse et al., 1998; Neuner and Buchner, 1999). Our estimations coincided with similar LT_50_ reported for *C. quitensis* with those methods (see **Table 2** for references). The electrolyte leakage method can lead to confusing results, because on one hand, it can overestimate leaf damage given that cellular solutes other than electrolytes may be induced by freezing, and on the other hand, coriaceous leaves do not release electrolytes readily, which can lead to spurious estimates of LT_50_ using this method (Boorse et al., 1998; Bannister, 2007). However, Antarctic vascular plants exhibited similar LT_50_ regardless they were calculated by ion leakage or photoinactivation methods as it is shown in the **Table 3**. In the case of *D. antarctica*, our LT_50_ values were intermediate compared to previous studies. Nevertheless, this result have to be taken with caution as some replicates did not reach the 50 percent damage (i.e., six replicates in the site 1). Thus, average LT_50_ in the field could be even more negative than we reported. This point is consistent with Casanova-Katny (1997) who found that the LT_50_ of *D. antarctica* was below -27°C in the field.

As mentioned, the ambient temperatures experienced by plants affect their ability to resist freezing temperatures (Beck et al., 2004). Thus, a second methodological aspect that differed among studies and that could underlie the discrepancies in the freezing resistance of Antarctic species is the residence time and temperature used on growth chambers. For example, Bravo et al. (2001) and Gianoli et al. (2004) collected adult plants from Antarctica that were vegetative propagated at 15°C for a couple of years before the freezing determinations. Chew et al. (2012) obtained adult plants from seeds collected in the field, while Reyes-Bahamonde (2013) used plants grown at constant 11°C during 2 months after their collection in the field. However, although Bravo et al. (2001) and Gianoli et al. (2004) used similar plants residence time and growth temperature for determinations, they found different LT_50_ for *C. quitensis.* A similar situation occurred for *D. antarctica* (**Table 2**). Probably, multiple factors could influence the level of freezing resistance. Such discrepancies reinforce the importance of *in situ* determinations as we did here.

Finally, our results clearly showed that warmer temperatures affect the freezing resistance of Antarctic vascular plants. These results are in line with previous studies conducted in alpine and arctic plant where similar plant responses to warming (decreases in freezing resistance with warming) were reported (e.g., Loveys et al., 2006; Marchand et al., 2006; Woldendorp et al., 2008; Sierra-Almeida and Cavieres, 2010; Rixen et al., 2012). The ability to withstand freezing temperatures is a key feature of species inhabiting cold climates, hence if new warming phases occur in the Antarctic Peninsula due to climate change, the survival of *C. quitensis* could be threatened. Whilst *D. antarctica* seems to be unaffected by warming on this trait, other aspects of its biology could be altered by warming (e.g., increases in respiration). Nevertheless, more research is needed to unveil the likely consequences of global warming on plants from cold biomes where *in situ* determinations of plant freezing resistance are crucial to understand the physiological mechanisms underlying plant adaptations to current and future climatic scenario for the Antarctic in particular.

## Author Contributions

AS-A conceived and designed the study, conducted the data collection, analyzed the data, and edited the manuscript. LC contributed to the study conception, designed and installed experimental setup and climatic sensors, and edited the manuscript. LB designed and installed experimental setup and climatic sensors, and contributed to the data analysis and manuscript approval.

## Conflict of Interest Statement

The authors declare that the research was conducted in the absence of any commercial or financial relationships that could be construed as a potential conflict of interest.
