# Supplementary material for: Warmer Temperatures Affect the in situ Freezing Resistance of the Antarctic Vascular Plants
Source: Front Plant Sci. 2018 Oct 8;9:1456. doi: 10.3389/fpls.2018.01456 (PMC6187968; doi:10.3389/fpls.2018.01456)
Supplement: Supplementary file 1 [file Table_1.DOCX]

Supplementary Material

Warmer temperatures affect the *in situ* freezing resistance of the Antarctic vascular plants

**Angela Sierra-Almeida^*^, Lohengrin A. Cavieres, León A. Bravo**

*** Correspondence:** Angela Sierra-Almeida: angelasierra@udec.cl

**Table S1.** Soil nutrients obtained from samples collected early in the first growing season (2013-2014) for the three studied sites. Values correspond to mean ± standard error (*n* =3−4). Different lower cases indicate significant differences after Kruskall-Wallis ANOVA by ranks and post-hoc comparisons (*P* <0.05).

| Component | **Site 1** | **Site 2** | **Site 3** |
| --- | --- | --- | --- |
| N (mg kg^-1^) | 38 ± 6.4^a^ | 17.7 ± 1.5^b^ | 4.0 ± 0^c^ |
| P (mg kg^-1^) | 268.3 ± 10.1^a^ | 301.7 ± 46.9^a^ | 19.0 ± 17.6^b^ |
| K (mg kg^-1^) | 170.7 ± 32^a^ | 487.4 ± 70^b^ | 268.2 ± 41.2^c^ |
| pH (in water) | 4.9 ± 0^a^ | 5.3 ± 0.1^b^ | 6.9 ± 0.3^c^ |
| Organic matter (%) | 9.2 ± 2.2^a^ | 5.7 ± 0.3^b^ | 1.2 ± 0.1^c^ |

**Table S2.** ANOVA tables for the effects of warmer temperatures (presence of OTC) and site on the ice nucleation temperature (NT, °C), freezing point (FP, °C) and low temperature damage (LT_50_, °C) measured in *Colobanthus quitensis* and *Deschampsia antarctica* plants during the growing season 2015 in the King George Island (Antarctic Peninsula).

| **Effect** | **SS** | **df** | **MS** | **F** | ***P*** |
| --- | --- | --- | --- | --- | --- |
| *C. quitensis* |  |  |  |  |  |
| **NT** |  |  |  |  |  |
| Warming | 0.080 | 1 | 0.080 | 6.82 | 0.014 |
| Site | 0.028 | 2 | 0.013 | 1.18 | 0.320 |
| Interaction | 0.010 | 2 | 0.005 | 0.43 | 0.655 |
| Error | 0.375 | 32 | 0.012 |  |  |
|  |  |  |  |  |  |
| **FP** |  |  |  |  |  |
| Warming | 0.031 | 1 | 0.031 | 2.62 | 0.012 |
| Site | 0.097 | 2 | 0.048 | 4.10 | 0.026 |
| Interaction | 0.031 | 2 | 0.015 | 1.30 | 0.286 |
| error | 0.377 | 32 | 0.012 |  |  |
|  |  |  |  |  |  |
| **LT_50_** |  |  |  |  |  |
| Warming | 21.213 | 1 | 21.213 | 34 | <0.0001 |
| Site | 215.282 | 2 | 107.64 | 172.53 | <0.0001 |
| Interaction | 3.366 | 2 | 1.683 | 2.70 | 0.083 |
| error | 19.965 | 32 | 0.624 |  |  |
|  |  |  |  |  |  |
| *D. antarctica* |  |  |  |  |  |
| **NT** |  |  |  |  |  |
| Warming | 0.032 | 1 | 0.032 | 5.42 | 0.026 |
| Site | 0.280 | 2 | 0.140 | 24.13 | <0.0001 |
| Interaction | 0.051 | 2 | 0.026 | 4.42 | 0.020 |
| error | 0.198 | 34 | 0.006 |  |  |
|  |  |  |  |  |  |
| **FP** |  |  |  |  |  |
| Warming | 0.141 | 1 | 0.141 | 8.40 | 0.007 |
| Site | 0.408 | 2 | 0.204 | 12.17 | <0.001 |
| Interaction | 0.041 | 2 | 0.021 | 1.23 | 0.306 |
| error | 0.569 | 34 | 0.017 |  |  |
|  |  |  |  |  |  |
| **LT_50_** |  |  |  |  |  |
| Warming | 2.21 | 1 | 2.21 | 1.06 | 0.312 |
| Site | 95.46 | 2 | 47.73 | 22.75 | <0.0001 |
| Interaction | 28.3 | 2 | 14.15 | 6.74 | 0.003 |
| error | 71.34 | 34 | 2.10 |  |  |

**Table S3.** Level and mechanism of freezing resistance of Antarctic plant species studied in the King George Island (Antarctic Peninsula). Field determinations were conducted in leaf of plants growing under natural temperature (-W) and warming (+W) conditions, at three sites in the study area. Freezing resistance parameters (°C): NT, ice nucleation temperature; LT_50_, temperature producing 50% damage; and thermal difference between LT_50_ and NT. Data correspond to mean values ± SE. Statistical comparisons between NT and LT_50_ to determine mechanisms: FT, freezing tolerance; FA, freezing avoidance are shown as level of significance: **P* <0.01; ** *P* <0.001; and *** *P* <0.0001.

|  | ***Colobanthus quitensis*** | | ***Deschampsia antarctica*** | |
| --- | --- | --- | --- | --- |
| **Site 1** | **control** | **warming** | **control** | **Warming** |
| NT | -3.6 ± 0.3 | -4.3 ± 0.6 | -3.6 ± 0.3 | -4.4 ± 0.2 |
| FP | -2.1 ± 0.2 | -2 ± 0.1 | -2.5 ± 0.3 | -3.1 ± 0.2 |
| LT_50_ | -17.4 ± 0.5 | -15.7 ± 0.2 | -24 ± 1 | -24.9 ± 0.03 |
| Thermal difference | 13.8** | 11.4** | 20.4*** | 19.8** |
| mechanism | FT | FT | FT | FT |
| **Site 2** |  |  |  |  |
| NT | -3.8 ± 0.2 | -5.3 ± 0.7 | -5.2 ± 0.4 | -4.7 ± 0.3 |
| FP | -2.4 ± 0.2 | -3 ± 0.4 | -3.2 ± 0.3 | -3.5 ± 0.3 |
| LT_50_ | -12.4 ± 0.1 | -10.2 ± 0.4 | -24.1 ± 0.2 | -21.3 ± 0.1 |
| Thermal difference | 8.6*** | 4.9** | 18.9** | 16.6** |
| mechanism | FT | FT | FT | FT |
| **Site 3** |  |  |  |  |
| NT | -3.5 ± 0.1 | -4.4 ± 0.3 | -5.6 ± 0.4 | -7.5 ± 0.6 |
| FP | -2 ± 0.2 | -2.6 ± 0.2 | -4 ± 0.6 | -6.1 ± 0.6 |
| LT_50_ | -16 ± 0.3 | -15.4 ± 0.1 | -20.4 ± 0.8 | -20.9 ± 0.5 |
| Thermal difference | 12.5 * | 11* | 14.8*** | 13.4*** |
| mechanism | FT | FT | FT | FT |
